# Section 12 approval: fit for purpose?

**DOI:** 10.1192/bjb.2020.39

**Published:** 2020-06

**Authors:** Nidhi Gupta, William Calthorpe

**Affiliations:** ST5, South Assertive Outreach Team, Birmingham and Solihull Mental Health Foundation Trust, email: nidhigupta@doctors.net.uk; Consultant Psychiatrist, South Assertive Outreach Team, Birmingham and Solihull Mental Health Foundation Trust

We compliment Rigby and McAlpine on a well-written editorial on Section 12 approval.^1^ The authors raise several pertinent issues about Mental Health Act (MHA) detentions and note that there has been a 47% increase in the rate of detentions countrywide. They are of the opinion that the increase in detentions is mainly attributable to clinicians not being equipped with the necessary knowledge and training. Rigby and McAlpine suggest more rigorous evidence-based training reinforced by appropriate assessment, including summative assessment using criteria-referenced methods with pass marks determined by the Angoff method. The authors also feel that the approval and revalidation processes need to be more robust.

We, however, are of the opinion that the authors have taken an Occam's razor view by largely attributing the problem to clinicians’ training. In our opinion, increases in detention rates are due to multiple factors, and the ‘fix’ is not as binary as upgrading training of clinicians or making the approval and revalidation processes more robust. The process of MHA assessment requires two doctors, of which one has to be an independent Section 12 doctor, and an approved mental health professional (AMHP), who is usually (but not invariably) a social worker. All three have to agree to detain a patient with a mental disorder. It is pertinent that the final responsibility for detaining someone under an MHA belongs to the AMHP, who then submits an application to a local hospital for an in-patient bed.

We quote verbatim from the 2018 Care Quality Commission report^2^ on the use of the MHA to detain people:

‘1. The apparent rise in rate of detention since 2010 is in part due to the national data return being more complete.

2. More people are being detained on more than one occasion during a calendar year than was previously the case.

3. Bed numbers have fallen and more people with severe mental health problems are living outside of a hospital setting, and so are at greater risk of being detained.

4. Some people are being detained under the MHA who would previously not have been detained. This is because clinicians are applying the criteria for detention differently to people with certain types of disorder (such as dementia or personality disorder). It could also be because more people with mental health problems are coming to the attention of mental health care workers (for example, through schemes that divert people from the criminal justice system).

5. People who need admission and who would previously have agreed to informal admission are now refusing and are being admitted as detained patients.

6. Admissions (some of which would be formal) that could in the past have been prevented are now not being prevented because less restrictive alternatives in the community are not available.

7. There has been an increase in the total size of the population of England and an increase in the size of those sections of the population that are more at risk of detention.

8. There has been an increase in the prevalence of risk factors for detention, such as social exclusion and problematic, untreated drug and alcohol misuse.’

Glover-Thomas, in a recent review, notes that the availability of mental health beds has decreased, thereby delaying the ‘preferred option’ of voluntary admission of patients. Therefore, in circumstances when clinicians deem a patient to be in need of care in hospital, resorting to detention ‘may be the quickest means of opening up services’.^3^ This factor – in our view – is consequential in ‘bumping up’ detention rates.

The number of appeals to mental health review tribunals (MHRTs) in England and Wales has risen steadily, from 904 in 1980^4^ to 31 469 in 2014.^5^ This reflects the parallel increase in the number detained. However, the percentage of patients who are successful in obtaining discharge at MHRT hearings is relatively low (only 9% of all hearings in 2013–2014 resulted in discharge^6^), suggesting that patients have been detained appropriately. This, in turn, suggests that training for Section 12 approval is not a factor. However, we agree with Rigby and McAlpine that improvements in training would be beneficial to clinicians in terms of increasing their confidence and knowledge.
